# Experimental Models of Infectious Pulmonary Complications Following Hematopoietic Cell Transplantation

**DOI:** 10.3389/fimmu.2021.718603

**Published:** 2021-08-16

**Authors:** Xiaofeng Zhou, Bethany B. Moore

**Affiliations:** ^1^Dept. of Microbiology and Immunology, University of Michigan Medical School, Ann Arbor, MI, United States; ^2^Division of Pulmonary and Critical Care Medicine, Dept. of Internal Medicine, University of Michigan Medical School, Ann Arbor, MI, United States

**Keywords:** hematopoietic cell transplantation, bone marrow transplantation, infectious pulmonary complications, herpesvirus, *Pseudomonas aeruginosa*, *Aspergillus fumigatus*

## Abstract

Pulmonary infections remain a major cause of morbidity and mortality in hematopoietic cell transplantation (HCT) recipients. The prevalence and type of infection changes over time and is influenced by the course of immune reconstitution post-transplant. The interaction between pathogens and host immune responses is complex in HCT settings, since the conditioning regimens create periods of neutropenia and immunosuppressive drugs are often needed to prevent graft rejection and limit graft-*versus*-host disease (GVHD). Experimental murine models of transplantation are valuable tools for dissecting the procedure-related alterations to innate and adaptive immunity. Here we review mouse models of post-HCT infectious pulmonary complications, primarily focused on three groups of pathogens that frequently infect HCT recipients: bacteria (often *P. aeruginosa*), fungus (primarily *Aspergillus fumigatus*), and viruses (primarily herpesviruses). These mouse models have advanced our knowledge regarding how the conditioning and HCT process negatively impacts innate immunity and have provided new potential strategies of managing the infections. Studies using mouse models have also validated clinical observations suggesting that prior or occult infections are a potential etiology of noninfectious pulmonary complications post-HCT as well.

## Introduction

Hematopoietic cell transplantation (HCT) is a potentially curative treatment for high-risk hematopoietic neoplastic disorders, metabolic, genetic and immune-mediated diseases. It involves eradication or suppression of the recipient’s hematopoietic cells using a conditioning regimen followed by infusion of stem cells collected from the bone marrow, placenta (cord blood) or peripheral blood (1). The source of hematopoietic cells can be either autologous (auto, recipient-derived) or allogeneic (allo, matched related or unrelated donor-derived) hematopoietic cells. HCT has been carried out increasingly over the years with 47,468 transplants in 50 European and associated countries (2), and 22,573 transplants in the United States in 2018 (3).

Unfortunately, the toxicity of conditioning regimens, alloimmune responses and immunosuppressive therapies cause severe post-transplant complications, in which the lung is one of the most common target organs. Pulmonary complications occur in up to 60% of allo-HCT recipients (4) and 25% of auto-HCT recipients (5). The frequent pulmonary complications and their significant contribution to post-transplant morbidity and mortality limit the success of HCT (6–8). These complications are heterogeneous, and include pathologies generated by infectious agents and noninfectious disorders. Although infectious pulmonary complications after HCT have been significantly reduced due to aggressive prophylaxis and the use of broad-spectrum antimicrobial medications, these infections still remain problematic, especially among the patients with graft-*versus*-host disease (GVHD). Major noninfectious pulmonary complications include early onset idiopathic pneumonia syndrome (IPS) (7), and late onset bronchiolitis obliterans syndrome (BOS) (9). Current experimental data support alloimmunity as an underlying mechanism of these idiopathic noninfectious lung injuries (7, 9). For a review on non-infectious pulmonary complications of stem cell transplantation, please see references (10, 11).

Animal models have been extensively used for the establishment and improvement of HCT therapy (12, 13). Animal models allow manipulation of single factors during the development of complications associated with HCT, and thus are crucial for successfully improving clinical applications. Most of the current knowledge regarding defects in immune responses during infectious pulmonary complications come from studies using mouse models of HCT. In this review, we will first briefly introduce infectious pulmonary complications post-HCT, and then describe relevant mouse models and current understanding of host immune responses to lung infections post-HCT that have been acquired from studying these models.

## Clinical Phenotypes Of Infectious Pulmonary Complications Post-Hct

The immune system of HCT recipients is eradicated or weakened by either myeloablative or less intense nonmyeloablative conditioning regimens before transplant to eradicate/reduce tumor burdens and to prevent graft rejection. Thus, it is not surprising that infections are a major complication post-HCT. Infectious complications are more frequent and severe in patients with allo-HCT due to prolonged immunosuppressive therapy and GVHD (14). The timing of reconstitution of the immune system post-HCT varies considerably among patients, depending on the type of transplant (autologous *vs* allogeneic), the intensity of conditioning regimen, the source of hematopoietic cells, the presence of GVHD and the length of immunosuppressive therapies. Nevertheless, post-HCT reconstitution can be roughly divided into three phases: severe neutropenia or pre-engraftment phase (first 2-4 weeks), early engraftment phase (second and third month) and late engraftment phase (after second or third month) (14).

The prevalence and types of infection change over time and often follow the course of immune reconstitution post-transplant in patients (14). During the pre-engraftment phase, the depletion of neutrophils and damage to the mucosal barriers caused by conditioning regimens allow opportunistic pathogens to become infectious. The predominant pathogens during this phase are *Pseudomonas, Candida* and *Aspergillus* species (15–18). During the early engraftment phase, most innate immune cell subsets such as monocytes, neutrophils, and natural killer cells repopulate at normal levels (19), but lymphocyte counts are still low. This allows the reactivation of herpesviruses, such as cytomegalovirus (CMV), Epstein–Barr virus (EBV), human herpesvirus 6 (HHV-6), and new infections with respiratory viruses (20–22). A second peak of invasive *Aspergillus* infection occurs at the end of the early engraftment phase in allo-HCT recipients due to prolonged GVHD and its immunosuppressive therapy (18). During the late posttransplant phase (about three months after transplant), innate immunity is mostly reconstituted, but the recovery of T cells takes about a year and B cells may take even longer to completely repopulate (23). Bacterial pneumonia is less common during this late phase, but allo-HCT recipients are still at risk of late CMV reactivation and fungal infection. Current preemptive therapeutic strategies have significantly reduced early onset CMV infections after allo-HCT, but the incidence of late CMV infections have increased (24, 25). CMV reactivation remains a life-threatening infectious complication that is difficult to manage following allo-HCT (26–28). Allo-HCT recipients with chronic GVHD and immunosuppressive therapy continue to be susceptible to *Aspergillus* and Gram-positive bacteria as well (29). It is thus important to understand the interplay among host immunity, pathogens and GVHD in an allo-HCT setting.

## Mouse Models Of Infectious Pulmonary Complications Following Bone Marrow Transplantation

There have been many functional studies on immune responses to pathogens in mouse models of HCT. These have included both syngeneic (syn) and allogeneic strain combinations to recapitulate autologous or allogeneic HCT in patients. The pneumonia pathogens studied span bacteria (mostly *Pseudomonas aeruginosa*), fungus (primarily *Aspergillus fumigatus*), and viruses (primarily herpesviruses). There are also reports of sepsis subsequent to gastrointestinal damage due to conditioning regimens in HCT mouse models (30).

### Mouse Models of Post-HCT Bacterial Pneumonia

Bacterial pneumonia usually occurs early after HCT during the neutropenic period (31), but can also occur post-engraftment. *P. aeruginosa* is the most common pathogen isolated from the lower respiratory tract within 100 days post-transplant (15). *P. aeruginosa* is a ubiquitous environmental bacterium, and if inhaled into the lung airway by a immunocompetent individual, it is quickly cleared by alveolar macrophages (AMs) (32). Because *P. aeruginosa* has become increasingly resistant to multiple antibiotics over the years (33), it can be difficult to treat multidrug resistant *Pseudomonal* pneumonia (MDRPa) (16). About 40% of the hematologic malignancy patients infected with MDRPa will die in 30 days (34) and MDRPa outbreaks are associated with a death rate as high as 80% (35).

**Figure 1:**
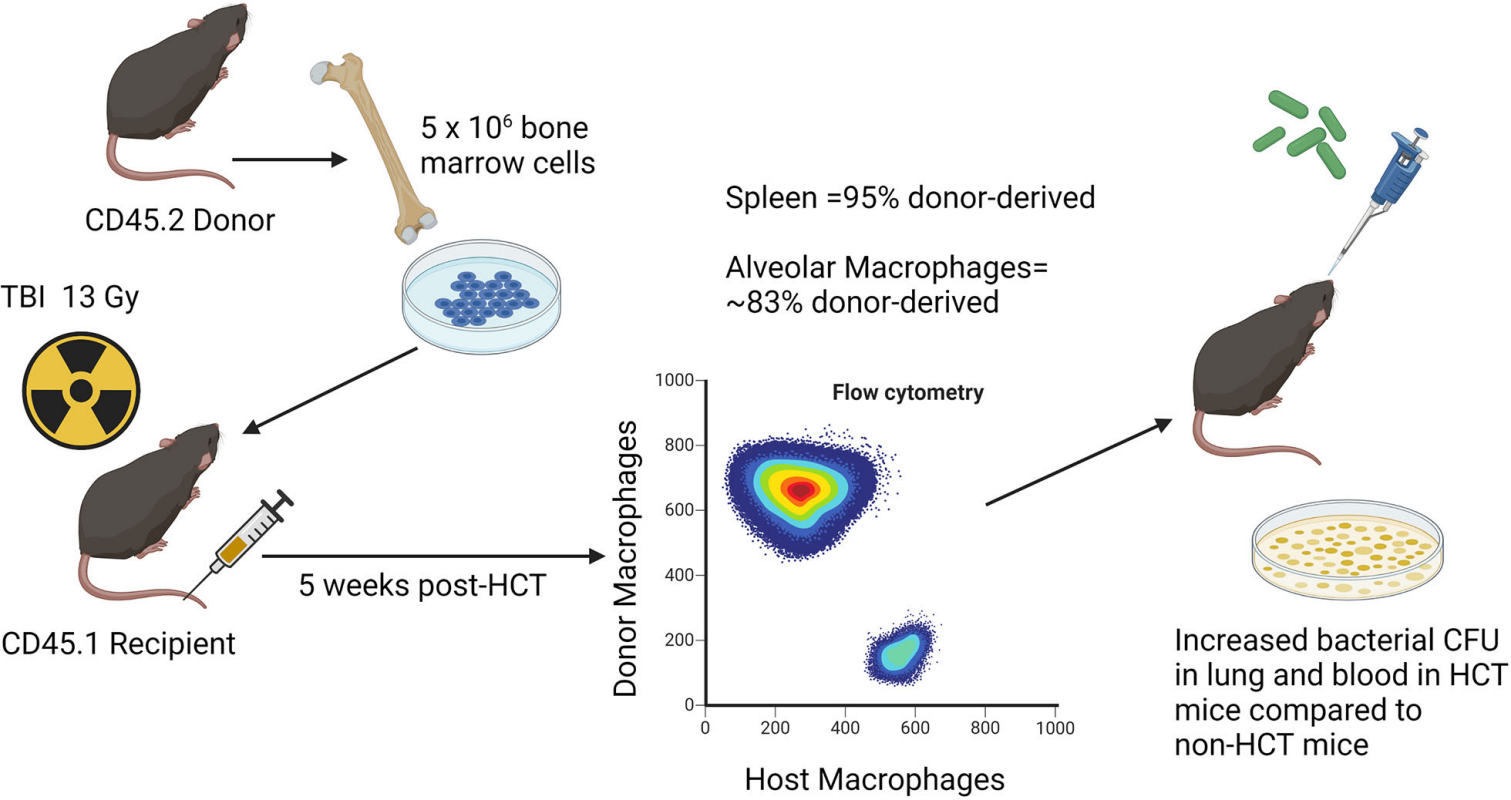
Schematic illustration of syn HCT mouse model. The figure shows the process of syn HCT used in experiments to test impaired host defense against *Pseudomonas aeruginosa* as described in the text. The figure is created with Biorender.com.

A syn-HCT mouse model was established in our laboratory to understand why HCT recipients are susceptible to *P. aeruginosa* (36). This model is clinically relevant, as both autologous and allogeneic transplant patients are susceptible to *P. aeruginosa* infection (37). Like auto-HCT recipients, mouse syn-HCT recipients have no risk of GVHD, and thus the model can be used to explore how the transplant procedure alone impacts pulmonary immunity. Recipient C57BL/6 mice are given a split dose of 13 Gy total body irradiation (TBI) from either a ^137^Cs or x-ray orthovoltage source with an interval of 3 hours between doses. Bone marrow is harvested from donor C57BL/6 mice, and 5x10^6^ whole bone marrow cells are infused into the recipients *via* tail vein injection. Five weeks after transplant, the percentage of donor-derived cells is approximately 95% in the spleen and the percentage of donor-derived AMs in the lung is about 83% (38). At this time point, HCT or age-matched non-HCT control mice are infected with *P. aeruginosa* PAO1 *via* intratracheal (i.t.) inoculation (36). These experiments demonstrated increased bacterial burden in the lung and dissemination to the blood at 24 h post-infection in HCT mice compared to non-transplant controls (36). See **Figure 1** for schematic illustration of the model system.

The defect in bacterial clearance in HCT mice is associated with reduced phagocytosis and killing of *P. aeruginosa* in lung AMs (39, 40) and impaired killing and defective formation of neutrophil extracellular traps (NETs) in neutrophils (41). Similar to HCT patients, the levels of immunosuppressive prostaglandin E_2_ (PGE_2_) are elevated in HCT mice (39, 42). Subsequent studies found that overproduction of PGE_2_ impairs the functions of both AMs and neutrophils, and pharmacologic inhibition of PGE_2_ production *in vivo* restores host defense of HCT mice (39, 41).

The syn-HCT model permits further dissection of the mechanisms explaining how the HCT procedure promotes AMs to overproduce PGE_2_. Conditioning-associated cellular stress stimulates alveolar epithelial cells to produce TGF-β (43). TGF-β signaling stimulates AMs to transcribe microRNA (miR)-29b which suppresses the expression of DNA methyltransferases (DNMTs) (44). Under homeostatic conditions, DNMT3a and DNMT3b methylate the promoter region of cyclooxygenase (COX)-2 gene which encodes a critical enzyme for the production of PGE_2_ (40). Methylation of the COX-2 promoter limits transcription and reduces COX-2 gene expression. Suppression of DNMTs by the TGF-β-miR-29b axis releases this endogenous break on COX-2 expression and thus increases the production of PGE_2_ in AMs (44). Interestingly, these epigenetic changes can be long lived with human HCT patients (even 6 years post-HCT) showing elevated levels of PGE_2_ in the lung. Thus, this mechanism likely accounts for long-lived innate immune impairment post-HCT.

**Figure 2:**
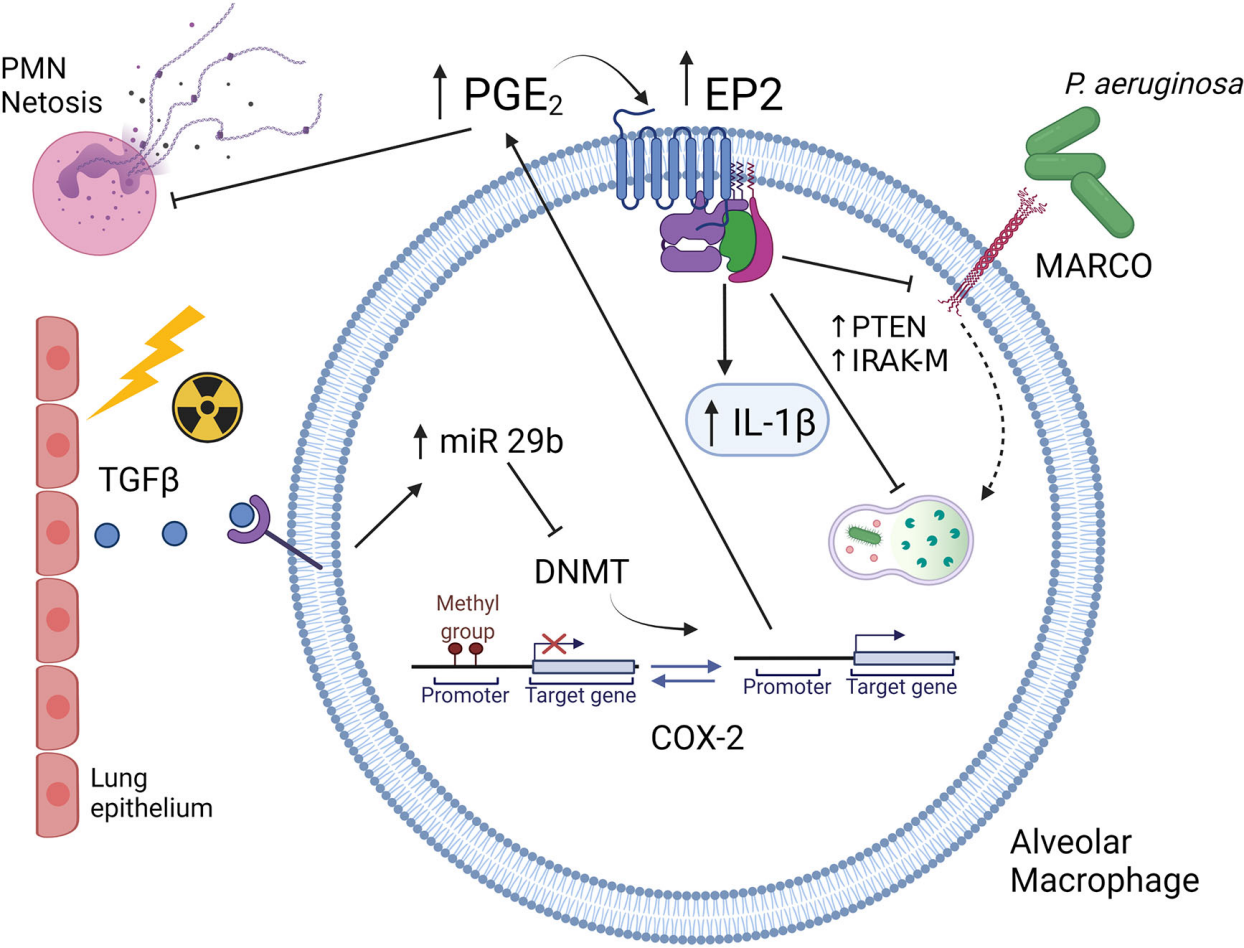
Schematic illustration of Syn HCT induced changes in innate immunity. Conditioning with TBI causes injury to lung epithelium resulting in production of TGF-β. Binding of TGF-β to alveolar macrophages results in increased miR29b expression which then limits expression of DNA methyltransferases (DNMT). This allows for the promoter of the cyclooxygenase 2 (COX-2) gene to be unmethylated resulting in increased production of prostaglandin E2 (PGE_2_). PGE_2_ then binds to the E prostanoid 2 (EP2) receptor which is also upregulated post-HCT. Downstream signaling by PGE_2_ results in upregulation of IL-1, elevations in phosphatase and tensin homolog on chromosome 10 (PTEN) and elevations in IL-1 receptor associated kinase (IRAK-M). These changes impair intracellular killing while downregulation of the MARCO scavenger receptor impairs phagocytosis of *P. aeruginosa.* Original references described in text. The figure is created with Biorender.com.

The immunosuppressive function of PGE_2_ is mediated by its receptors E prostanoid receptor 2 (EP2) and EP4 (44). Signaling *via* these receptors can activate a cyclic adenosine monophosphate (cAMP)-mediated signaling cascade with multiple downstream effects. One effect is downregulation of the scavenger receptor MARCO which is critical for recognition and phagocytosis of *P. aeruginosa* (45). Another effect is upregulation of IL-1 receptor associated kinase M (IRAK-M), which is an inhibitor of TLR signaling (46). Ultimately, this alteration impairs the proinflammatory cytokine response (e.g. TNF-α and IFN-γ) that could help clear bacterial infection. At the same time, PGE_2_ promotes transcription of IL-1β which is a mediator of tissue damage in the lung (47). Furthermore, PGE_2_ stimulates the expression of phosphatase and tensin homolog deleted on chromosome 10 (PTEN), which negatively regulates phagocytosis and killing of *P. aeruginosa* (48). A summary figure describing some of the innate immune changes in AMs post-HCT is found in **Figure 2**.

In contrast to the impaired phagocytosis of *P. aeruginosa* post-HCT by AMs, phagocytosis of *Staphylococcus aureus* is actually enhanced (45). This increased phagocytosis of *S. aureus*, is also regulated by PGE_2_, by stimulating the expression of miR-155 which upregulates scavenger receptor (SR-)AI/II (45). However, despite the enhanced uptake of *S. aureus*, AMs from HCT mice are unable to effectively kill the pathogen intracellularly as a result of the impacts on IRAK-M and PTEN described above. Additionally, the impaired innate immune function of neutrophils likely contributes to poor *S. aureus* clearance. Interestingly, a study by Zimecki et al. explored the use of bacteriophages as a therapeutic strategy for syn-HCT mice infected with *S. aureus* strain L (49). Similar to the findings reported above, HCT mice were highly susceptible to *S. aureus* infection (only 8.3% of infected mice survived whereas mice treated with phage showed 72% survival. It was also noted that the phage therapy increased the circulating leukocyte and neutrophil counts.

While the majority of the studies reviewed above show defective innate immune function, there is one study from 1989 showing that early after marrow transfer in allogeneic radiation chimeras that macrophages can be non-specifically activated by the conditioning milieu and show enhanced resistance to *Listeria monocytogenes* initially, but that this protection eventually declines with time (50).

### Mouse Models of Post-HCT Fungal Pneumonia

Invasive fungi have become the leading infectious cause of morbidity and mortality in HCT recipients in the era of improved prophylaxis and treatment of bacterial and viral infections (51, 52). Invasive pulmonary aspergillosis (IPA) is the most common fungal infection in the lung of HCT recipients. IPA causes high mortality among HCT patients, ranging from 30% to 70% (53), accounting for 10% of all death among the recipients (54). IPA is highly associated with neutropenia, GVHD and its related immunosuppressive therapy, and thus the incidence of IPA peaks early in the pre-engraftment phase and then later post-engraftment in allo-HCT recipients (18). This bimodal distribution of IPA post-HCT reflects different etiologies of *Aspergillus* infection: early IPA is due to prolonged neutropenia, especially when using myeloablative conditioning regimens, while late IPA is secondary to receiving corticosteroid or other immunosuppressive therapies to treat GVHD (55). Interestingly, the pro-inflammatory status noted in allo-HCT mice without immunosuppressive treatment may enhance clearance of *Aspergillus* as noted in one study by Hildebrandt et al. (56). The significant shift to using lower intensity non-myeloablative regimens allows a shorter neutropenic period and the systematic use of antifungal prophylaxis has led to a decrease in the incidence of early IPA (57). The early and late IPA time periods present distinct immunopathology patterns in HCT recipients. Early IPA in the neutropenic phase is characterized by rapid fungal growth and low levels of inflammation, but late IPA in immunosuppressed patients usually presents with overabundant inflammation including excessive neutrophil infiltration with insufficient fungal clearance (58).

Accordingly, several animal models have been established to understand the pathogenesis of early or late aspergillosis in HCT patients (59). Neutropenic models include treating mice with chemotherapeutic agents such as cyclophosphamide (60), TBI (61, 62), and using antibody depletion of neutrophils (63). Immunomodulated models usually involve the use of corticosteroids (64). These two types of mouse models recapitulate the different pathologies of IPA that present in neutropenic and immunosuppressed patients respectively (64, 65). For the neutropenic mouse models, it is most common to administer cyclophosphamide at 150 mg/kg *via* the intraperitoneal route thrice weekly before infection. Some studies have used monoclonal antibodies to achieve neutrophil depletion. A dose of 100µg of anti-Ly6 (Gr1) rat IgG2b MAb57 (clone RB6-8C5) *via* intraperitoneal injection on the day before and 2 days after fungal intranasal inoculation dramatically reduces the number of neutrophils for up to 5 days (63). The lethal dose of irradiation varies depending on mouse strain receiving the treatment. For example, a single lethal dose of 9 Gy given to C3H/HeJ mice followed by transplants with 2x10^6^ T-cell-depleted allogeneic bone marrow cells from DBA/2 mice shows profound neutropenia 3 days after transplant (63). Immunomodulated models commonly administer cortisone at 100 to 200 mg/kg *via* subcutaneous injection thrice weekly for 1 or 2 weeks before experimental infection. In a cortisone-treated immunosuppressive model, myeloid cells such as neutrophils and macrophages, are massively recruited to the lungs upon infection, but lymphocytes fail to be recruited to the lung, indicating the requirement of lymphocytes to efficiently clear the infection (64). The most common routes of inoculation of *Aspergillus* are intranasal and intratracheal administration. A conidial suspension of *A. fumigatus* inoculated into the nares is close to natural infection, but due to upper mucociliary clearance of mice, only about 10% of the inoculum actually enters into the lungs (66). As a result, the development of IPA is highly variable in intranasally inoculated mice. Delivery of spore suspension directly into the trachea, by either tracheotomy or oropharyngeal aspiration in anesthetized mice, can more tightly control the fungal inoculum and lead to reproducible IPA (59). The most commonly used *A. fumigatus* strains are the low virulent strain AF293 (ATCC MYA4609 or CBS101355) and the high virulent strain Dal/CEA10 (ATCC MYA1163 or CBS 144.89). Depending on the strain of *A. fumigatus* and the route, the dose ranges from 1.0×10^2^ to 1.0×10^9^ conidia for mice infected through the intranasal route, and 1.0 to 2.0x10^7^ conidia through the intratracheal route (59).

**Figure 3:**
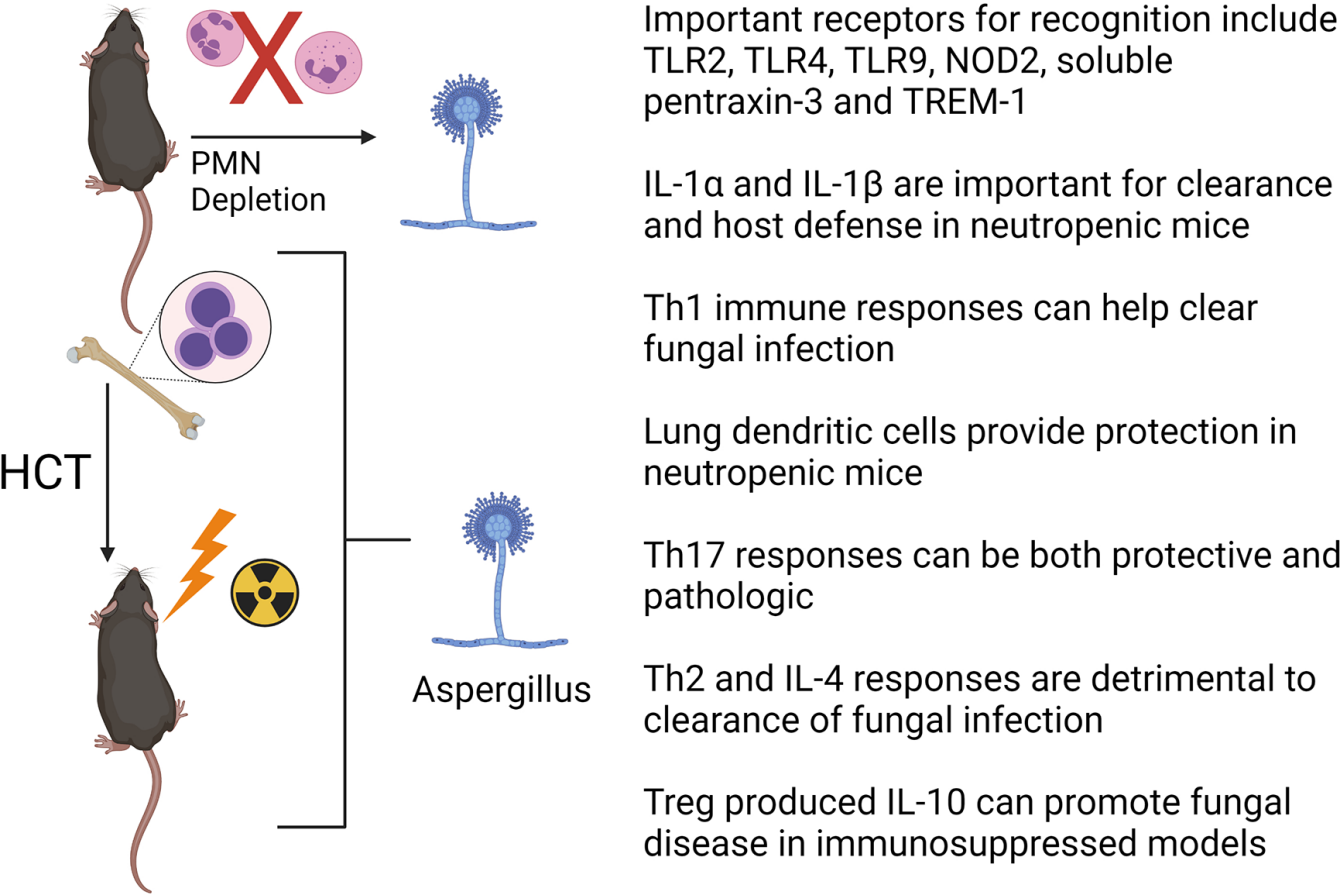
Factors important for clearance of *Aspergillus fumigatus* in HCT or neutrophil (PMN) depleted mouse models. See text for details. The figure is created with Biorender.com.

Studies on mouse models and human patients have greatly increased our knowledge of the host immune responses to *A. fumigatus* and have aided in the development of novel therapeutic targets to treat IPA [reviewed in references (67, 68)]. Here we highlight a few advances in the field during recent years. Mouse models have confirmed or identified several important pattern recognition receptors (PRRs) on the cell surface of innate immune cells, such as AMs, which include dectin-1 (61), TLR2 (69, 70), TLR4 (70), TLR9 (71–73), NOD2 (74), soluble pentraxin-3 (75) and TREM1 (76) in recognizing *Aspergillus* components. Inflammatory cytokines such as IL-1α and IL-1β are critical for host defense against *A. fumigatus* in neutropenic mice (77, 78). The role of antigen presentation and development of antigen specific T cell subsets has been studied with respect to clearance of IPA, but with some conflicting results. For example, adoptive transfer of dendritic cells pulsed with conidia stimulates a T helper type 1 (Th1) responses and improves survival in a syn-HCT model suggesting an important role of Th responses to clear infection (79). This is consistent with several other studies in mouse HCT models that also demonstrated that Th1-mediated immunity is important in clearing *A. fumigatus* infection (80–82). Interestingly, it appears paradoxical that CCR7 deficient HCT mice whose dendritic cells cannot enter draining lymph nodes to prime T lymphocytes show improved survival in a monoclonal antibody (anti-Gr1) induced neutropenic model (83). This study suggests that retaining CD11b+ dendritic cells inside the neutropenic lung during initial infection with *Aspergillus* is beneficial, potentially by complimenting the loss of neutrophils. The role of the Th17 response in the pathogenesis of IPA is somewhat controversial, as it can be either protective (84, 85) or pathogenic (80). Th2 responses and the production of IL-4 are detrimental to control *A. fumigatus* infection, as IL-4^-/-^ mice are protected from IPA (86). Finally, regulatory T cells are producers of IL-10 which is linked to disease progression in steroid immunosuppressive experimental IPA (87, 88). **Figure 3** provides a summary of recent insights from mouse models of HCT or neutropenia with regards to Aspergillus infection.

### Mouse Models of Post-HCT Viral Pneumonia

Viral infections often occur after engraftment when the reconstitution of lymphocytes is not yet complete, or when immunosuppression due to prophylaxis or treatment of GVHD in allo-HCT recipients is needed. Nearly every human being is infected with one or more herpesviruses in the first two decades of life and the viruses can establish life-long latency to escape immune surveillance and detection (20). Viral pneumonia can be caused by reactivation of latent human herpesviruses or new infection with community acquired respiratory viruses. Additionally, primary herpesvirus infections can occur in seronegative patients receiving grafts from seropositive donors (89). Besides causing direct lung injuries, such as, cytomegalovirus (CMV) pneumonia (20), occult or prior herpesvirus infections appear to trigger the development of “noninfectious” pulmonary complications at later time points after allo-HCT (21, 22, 90).

Most human herpesviruses have very strict host-species specificity, and it is thus difficult to study human herpesviruses in mice. Herpes simplex virus type 1 (HSV-1) is an exception, as it can directly cause pneumonia in HCT mice (91). To bypass this hurdle, some researchers have generated transgenic mouse models that express receptors for human herpesvirus. For example, human CD46, an HHV-6A receptor, is expressed in the brain of a mouse line to study host innate immunity against HHV-6A (92). Other researchers engrafted human CD34+ hematopoietic progenitor cells into NOD-*scid IL2Rγc^null^* (NSG) mice which can then be directly infected with human herpesvirus (93). More often, murine homologs of their corresponding human herpesviruses are used in mouse models to study the principles of virus-host interactions that are thought to be shared among human and mouse systems. Mouse CMV (mCMV), murine gammaherpesvirus 68 (MHV-68) and murine roseolovirus (MRV) are frequently used to study human CMV, Epstein-Barr virus (EBV) and human herpesvirus (HHV)-6A/B, respectively in HCT settings.

Most murine herpesvirus models fall into two categories: pre-HCT latent infection or post-HCT infection models. Both syngeneic and allogeneic HCT have been studied. Latent infection models usually involve a primary infection in neonates or adult mice to mimic the natural history of herpesviral infection, followed by various lengths of “waiting time” to let the virus enter latency (21, 94). After that, an allo-HCT is usually performed to stimulate viral reactivation. Latently infected mouse models are most suitable for studying the reactivation of herpesvirus, their subsequent effects and for testing novel therapeutic strategies. This model has facilitated the discovery of the critical role of humoral immunity in controlling the reactivation of mCMV (94). The half-life of preexisting antibodies in latently infected mice and the elimination of recipient plasma cells due to GVHD can lead to a loss of anti-mCMV antibodies, which eventually leads to mCMV reactivation in recipients. Importantly, the reactivation of mCMV in allo-HCT recipients can be prevented by the transfer of immune serum (94). In a similar latent infection model, two doses of leukotriene B_4_ administered *via* intravenous route effectively reduced the reactivation of mCMV in allo-HCT mice through yet unknown mechanisms (95). To determine the relationship between herpesviral reactivation and noninfectious pulmonary complications, MRV, a mouse homolog of HHV-6, was given to neonatal mice and then reactivated in response to a minor histocompatibility antigen mismatched allo-HCT 8 weeks later. Indeed, the reactivation of MRV not only caused IPS-like pathology but also exacerbated histologic signs of acute GVHD in the gut (21).

Due to variable waiting times for entering latency and the heterogenous nature of post-HCT viral reactivation among latently infected mice, some researchers infect mice post-HCT or concurrent with HCT to mimic the reactivation of herpesvirus. The HCT procedure creates an immunosuppressive lung microenvironment, characterized by increased levels of PGE_2_ (39), TGF-β (91, 96) and Kynurenine (97). Reduced influx or altered function of CD8^+^ T cells, which are critical for clearance of mCMV (98, 99), HSV-1 (91) and community acquired respiratory viruses (100, 101), were observed in both syn- and allo-HCT recipients. As a result, most HCT mice experienced delayed viral clearance and persistent pneumonitis. Interestingly, the impact of HCT on T cell immunity does not seem to be mediated by the elevated levels of PGE_2_ (101), but rather by TGF-β (91).

Recently, studies using MHV-68, a mouse herpesvirus genetically related to Kaposi’s sarcoma-associated herpesvirus (KSHV) and EBV, in a syn-HCT model in our laboratory have advanced our understanding of host immune response to herpesvirus infection in the HCT setting. A C57BL/6 to C57BL/6 syn-HCT mouse model as described above was adopted to study MHV-68 infection. The HCT procedure not only causes an immunosuppressive environment, but also changes the structure of the lung microbiome (102). Together, these alterations in the lung microenvironment have significant impacts on the biology of conventional dendritic cells (cDCs) in HCT lungs. After exposure to MHV-68, the cDCs in syn-HCT mice increased their expression of pro-Th17 cytokines such as IL-6 IL-23 and TGF-β relative to the responses noted in untransplanted mice (103). These HCT lung cDCs also become deficient for delta like ligand 4 (DLL4), a Notch ligand, on their cell surface which further permits Th17 polarization (104). In addition, the migration of cDCs into mediastinal draining lymph nodes is impaired, significantly reducing Th1 responses, but augmenting Th17 responses which appear to be primed locally in the lung (105). Thus, the functional changes of lung cDCs post-HCT tip the balance of Th responses against MHV-68 infection from protective Th1 responses to pathogenic Th17 responses (96, 103).

**Figure 4:**
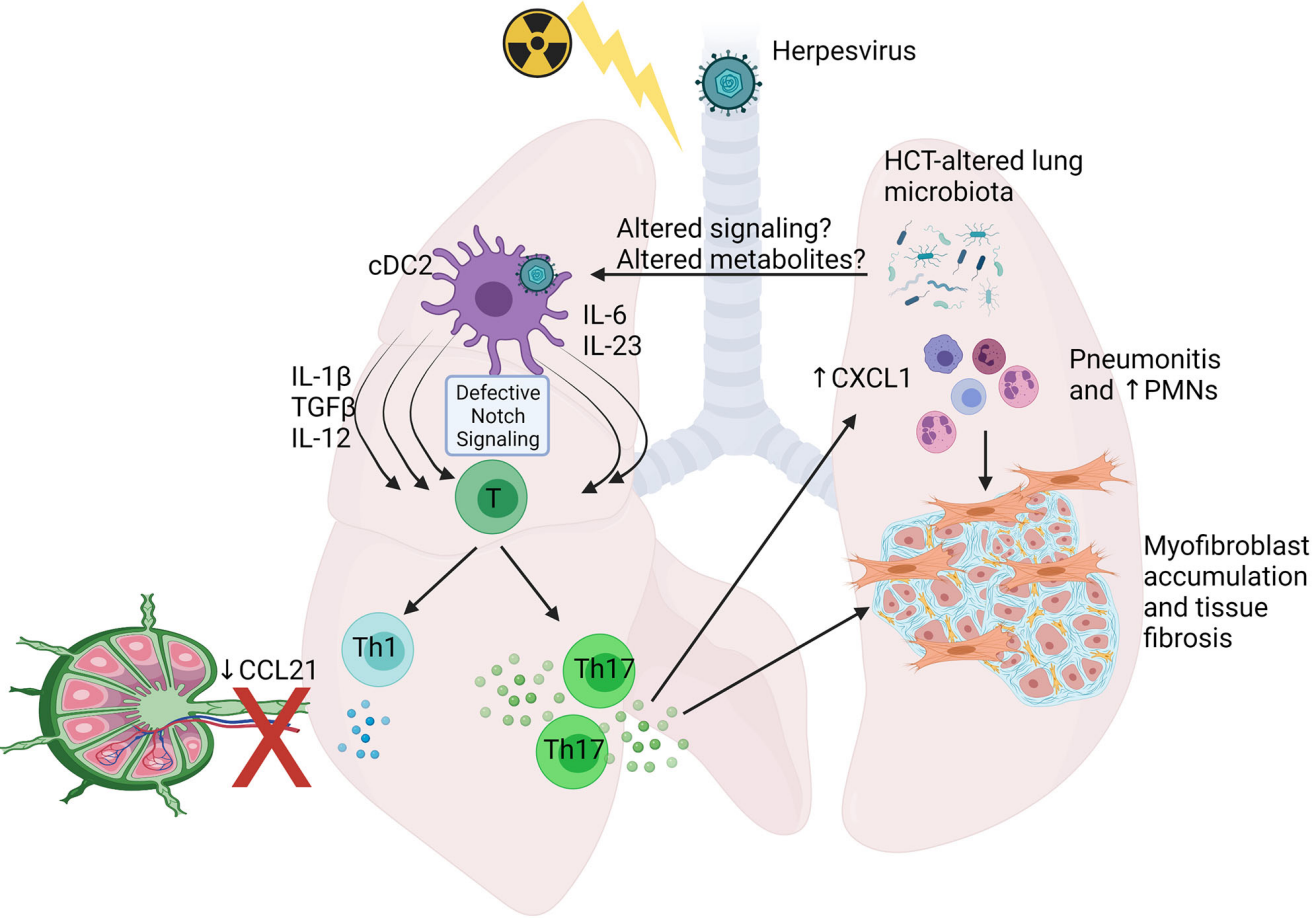
Schematic showing alterations shown to occur in Syn HCT mouse models of murine gammaherpesvirus infection. In responses to HCT, lung conventional dendritic cells 2 (cDC2) display defective delta like ligand 4 leading to impaired notch signaling when interacting with virus-specific T cells. This leads to production of cytokines able to drive both Th1 and Th17 cell differentiation, but because of poor ability of the cDCs to migrate to the draining lymph nodes secondary to reduced CCL21 levels, the Th1 response is impaired, while the Th17 response is primed efficiently in the lungs. Elevated IL-17 levels stimulate lung epithelium to produce CXCL1 and recruit PMNs prominently, although other cell types also accumulate. The IL-17 can also directly activate myofibroblast proliferation and extracellular matrix production leading to fibrosis. Alterations to the lung and gut associated microbiome are also prominent post-HCT and may contribute to altered immune cell functions. See text for details. The figure is created with Biorender.com.

Excessive IL-17A due to Th17 responses eventually causes the development of pneumonitis and pulmonary fibrosis 3 weeks after infection, when lytic MHV-68 is no longer detectable (103, 106). Administration of anti-IL-17A antibodies or using bone marrow cells isolated from IL-17A^-/-^ donor mice protects HCT recipients from pneumonitis and fibrosis after infection with MHV-68 (103). **Figure 4** highlights some of the changes noted in HCT lungs post-infection with herpesvirus. Note that the pathology seen in this mouse model also resembles many histological features seen in noninfectious complications such as IPS and restrictive lung disease, suggesting a potential etiology of noninfectious pulmonary complications caused by prior or occult infections that trigger pathogenic immune responses leading to lung injury and improper repair. This hypothesis is supported by recent discoveries of occult infections in IPS patients (90), and the strong association between the infections with herpesviruses (21) or community acquired respiratory viruses (22) and noninfectious complications in HCT recipients. Furthermore, direct evidence comes from a study mentioned above showing that reactivation of MRV in allo-HCT mice causes IPS-like pathology and exacerbates acute GVHD (21).

## Discussion

Pulmonary infections remain a major cause of morbidity and mortality in HCT patients. This is not surprising given that the conditioning regimens create periods of cytopenia and immunosuppressive drugs are often needed to limit GVHD. The rise of antibiotic resistant strains of bacteria, challenges of vaccinating immunocompromised patients, and limited availability of vaccines for many of the common pathogens in this patient population all help explain the infectious challenges facing HCT patients. Additionally, mucosal tissue damage as a result of GVHD and bacterial colonization can significantly increase the likelihood of opportunistic infection (107–109). As discussed above, non-myeloablative conditioning and fungal and viral prophylaxis have helped reduced infections during the cytopenic/neutropenic phases; however, there is a growing appreciation that even post-engraftment, innate and adaptive immune cells display altered immune function.

Even as early as 1982, it was noted that AMs from HCT patients were defective at phagocytosis, chemotaxis towards, and killing of fungal and bacterial pathogens when studied 4 months post-HCT (110). Importantly, while phagocytic and chemotactic defects normalized, the killing defects persisted even 12 months post-HCT (110). This suggests that alterations in the lung milieu, most likely caused by conditioning regimens may interfere with the function of innate immune cells. It also suggests that long-lived alterations may result from epigenetic alterations. A decade later, elevated levels of PGE_2_ were noted in both auto and allo-HCT patients; however, this finding was not linked to impaired innate immune function (42). It was not until 2006 that murine models were able to provide a mechanistic link between these two observations and demonstrate that overproduction of PGE_2_ post-HCT was responsible for impaired phagocytosis and killing by AMs (39). It took almost another decade to describe the epigenetic alterations in methylation of the COX-2 promoter that were caused by elevated levels of TGFβ1 caused by the conditioning regimen (44). Interestingly, this same mechanism that impairs innate immune function in HCT, has recently been shown to explain defective macrophage responses to wound healing and wound infection in diabetes as well (111). No doubt there are many other, yet to be discovered pathways that impair the function of innate immune cells in the transplant setting that are likely to be regulated *via* epigenetic alterations induced by the altered lung milieu.

We are also starting to learn more about how alterations of the normal microbiota in the lung and gut of HCT recipients may alter immune tone. The process of allo-HCT has been shown to reduce the diversity of the gut microbiota in humans (112). Furthermore, low diversity of gut flora at the time of neutrophil engraftment predicts mortality (113). Interestingly, when focused on the connection between gut microbiome and pulmonary complications, an observational study found that HCT patients that had low baseline gut microbiome diversity or proteobacteria domination early post-HCT had the highest incidence of pulmonary complications (114). This highlights the potential for a gut-lung axis when considering regulation of pulmonary immunity and such a concept has previously been suggested (115–118). More recently, the concept of alteration of the lung microbiome in the setting of HCT has demonstrated that dysbiosis is associated with alterations in inflammatory cytokines and poor outcomes (102, 119). Interestingly, in the murine studies, it was found that while the HCT procedure alone altered the microbiota, there was a more profound and prolonged alteration in the setting of herpesviral infection as well (102). Similarly, HCT patients with confirmed transcriptionally active pathogens in the bronchoalveolar lavage fluid display overall lower microbiome diversity in the lung, and many of these patients had viral infections (120). Whether the alterations caused by disturbances of the lung or gut microbiota are related to altered signaling *via* pathogen recognition receptors on immune cells or altered metabolites secreted by the microbiota or both remains to be determined. Interestingly, such findings may offer new diagnostic approaches. Zinter et al. recently performed metatranscriptomic analysis of pre-HCT bronchoalveolar lavage fluid in pediatric patients and found that children with evidence of viral enrichment and innate immune activation had the highest incidence of post-HCT lung injury while patients with diverse oropharyngeal taxa and lacking inflammatory signatures rarely developed post-HCT lung injury (119). While there are known differences in the composition of human and murine microbiota, murine models should still be useful for proof-of-concept studies regarding the role of potential prebiotics, fecal microbiome transplant and other potential therapies to improve outcomes post-HCT.

Our understanding of the host-pathogen interaction in HCT recipients has been accumulated over decades from studying animal models. Mice and humans have fairly similar organs and systems, immunity and pathology. Mouse models permit tightly controlled experimental conditions and unified genetics of host and pathogens. One of the most important advantages of mice is the availability of a huge collection of gene knockout or transgenic mouse strains, and it is now relatively easy to generate such mice if they are not readily available. In practical aspects, the cost of mice is inexpensive and experiments are reproducible. However, there are a few important limitations that need to be kept in consideration. First, the immune system in mice is considerably different from that in humans (121), and thus the knowledge acquired from mice may serve as “proof-of-concept”, but may not readily be translated to human clinical treatment. Second, mice are small and have short live spans. The small sizes of the body and lungs of mice may contribute to the different kinetics of immune reconstitution post-HCT and disease course in the lung compared with humans, as mouse lungs can be quickly overwhelmed by pathogens or immune cell infiltration. While there have been mouse models of BOS in mice that have provided important insights (122–128), it is not clear that the evolution of this disease in mice which have short lifespans fully recapitulate the features of disease that evolve over years in humans. Similarly, long term effects of chronic latency and reactivation of herpesviruses may be difficult to capture. Third, the host-pathogen interactions, especially with viral infections, is usually species-specific, which reduces clinical translatability. Other limitations of mouse models include a lack of parallel methodologies with the ones commonly used in clinical settings. Many of the studies have used syn-HCT models to avoid the complications of alloimmune responses, yet clinically, allo-HCT patients often have the most severe pulmonary complications and many of the current mouse models do not sufficiently explore the effects of GVHD or immunosuppressive therapy and how those factors impact immune function. Additionally, for convenience, mouse models often use TBI as the conditioning regimen, yet human HSCT is often accomplished with chemotherapy, reduced intensity irradiation or combinations. Despite these limitations however, the power of mouse genetics and the ability to genetically modify gene expression in a cell-type specific manner makes these murine models important tools that enable the dissection of fundamental mechanisms which underlie disease. Given that we do not have good anti-microbial strategies for many of the common pathogens that plague patients post-HCT, it is important to better understand how we can quickly repopulate the immune cells of the host and how we can manipulate the HCT regimens to improve the functionality of these immune cells. The power of HCT to cure inherited genetic diseases and malignancies will never be fully realized until the infectious complications, particularly in the lung can be better managed.

## Author Contributions

XZ and BM reviewed the literature, drafted the manuscript, created figures and edited the final document. All authors contributed to the article and approved the submitted version.

## Funding

Supported by R35HL144481 and DK124290.

## Conflict of Interest

The authors declare that the research was conducted in the absence of any commercial or financial relationships that could be construed as a potential conflict of interest.

## Publisher’s Note

All claims expressed in this article are solely those of the authors and do not necessarily represent those of their affiliated organizations, or those of the publisher, the editors and the reviewers. Any product that may be evaluated in this article, or claim that may be made by its manufacturer, is not guaranteed or endorsed by the publisher.
